# Assessment of Arterial Stiffness and Biochemical Markers in Systemic Lupus Erythematosus in the Diagnosis of Subclinical Atherosclerosis

**DOI:** 10.3390/jpm14030289

**Published:** 2024-03-08

**Authors:** Dominika Blachut, Brygida Przywara-Chowaniec, Michalina Mazurkiewicz, Andrzej Tomasik

**Affiliations:** 2nd Department of Cardiology, Medical University of Silesia in Katowice, 41-800 Zabrze, Poland

**Keywords:** systemic lupus erythematosus, atherosclerosis, prevention of cardiovascular diseases, pulse wave analysis, arterial stiffness, endothelial dysfunction

## Abstract

Patients with systemic lupus erythematosus (SLE) are 2–10 times more likely to develop cardiovascular disease (CVD) than the general population. The assessment of the risk of developing CVD is an important direction for further clinical management. The study was conducted retrospectively and included patients with SLE. The aim of the study was to assess the measurements of pulse wave velocity (PWV), carotid intima-media thickness (CIMT), ankle–brachial index (ABI) and biochemical parameters. Subclinical atherosclerosis was also assessed. The study included 98 patients with SLE with an age- and sex-matched control group of 68 healthy adults. Statistical significance was found in the SLE group and the controls for N-terminal fragment of pro-B-type natriuretic peptide (NT proBNP) (144.87 vs. 36.41 pg/mL, *p* = 0.0018), high-sensitivity cardiac troponin (hs-cTn) (25.43 vs. 6.38 ng/L, *p* = 0.0303) and D-Dimer levels (0.73 vs. 0.36 µg/mL, *p* = 0.0088), left CIMT (1.03 vs. 0.62 mm, *p* < 0.0001), right CIMT (0.93 vs. 0.63 mm, *p* < 0.0001) and PWV CF (9.74 vs. 7.98 m/s, *p* = 0.0294). A positive correlation was found between NT proBNP and PWV CF (r = 0.6880, *p* = 0.0498) and hs-cTn and PVW carotid-femoral (CF) (r = 0.8862, *p* = 0.0499) in SLE. A positive correlation was reported between PWV CF and systolic blood pressure (r = 0.5025, *p* = 0.0487). The measurement of carotid–femoral PWV is a simple, non-invasive, and reproducible method and may independently predict future CVD events and their cause. Further studies are warranted to establish the prognostic value of PWV in patients with SLE, as it may be superior to CIMT measurements in the early stages of vascular disorders.

## 1. Introduction 

Systemic lupus erythematosus (SLE) is a multisystem autoimmune disease of connective tissue. The incidence of SLE is estimated at 3–315 per 1,000,000, and the adjusted incidence is approximately 50–100 per 100,000. Over 60,000 (up to 80,000) cases of SLE were reported in Poland; the exact data are unknown [1,2,3]. Patients with SLE are 2–10 times more likely to develop cardiovascular disease (CVD) than the general population [4,5], and an increased predisposition to accelerated atherogenesis occurs at a young age [6]. Atherosclerosis and its complications are considered a cause of premature death in the SLE group, and SLE is regarded as an independent risk factor for the development of CVD [7,8]. Assessing the risk of CVD is an important direction for further clinical management. Endothelial dysfunction (ED) is one of the main causes leading to the development of atherosclerosis and its subsequent complications in the form of CVD in SLE. Such dysfunction is mainly associated with an imbalance between endothelial cell damage and repair in SLE patients [4,5]. Assessment and monitoring with non-invasive methods are key directions for CVD prevention. One of the parameters is carotid intima-media thickness (CIMT), acting as a surrogate marker of atherosclerosis. Several studies proved the presence of atherosclerosis in the pediatric population [9,10,11]. 

Another non-invasive test is the assessment of pulse wave velocity (PWV), which indirectly reflects arterial stiffness in the central circulation area and is an independent risk factor for CVD. Many studies confirmed that arterial stiffness is correlated with CVD events [12,13,14]. Arterial stiffness in SLE is associated with left ventricular dysfunction, left ventricular muscle hypertrophy or atherosclerosis in the main arteries [15,16]. The ankle–brachial index (ABI) is another non-invasive test to assess subclinical atherosclerosis. It is a specific predictor of CVD events [17,18]. An important element in the imaging assessment of the presence of atherosclerosis may be available markers for assessing the function of the circulatory system. High-sensitivity troponin is an important marker of acute coronary syndrome. Troponin release in SLE patients may be related to subclinical myocardial damage but also to the presence of atherosclerotic plaque with an apparently low risk of CVD [19,20,21]. The N-terminal fragment of pro-B-type natriuretic peptide (NT proBNP) is a marker of heart failure. NT proBNP is released from cardiac cells in response to volume and pressure overload. NT proBNP in SLE may correlate with organ dysfunction and the presence of atherosclerosis [22]. Correlations were observed between higher NT proBNP concentrations and the presence of changes in peripheral arteries [23,24]. In our study, the assessment of CVD risk in SLE was focused on surrogate markers of subclinical atherosclerosis, such as radiological and biochemical methods.

## 2. Materials and Methods

The study was conducted retrospectively and included patients of the Second Department of Cardiology and the Cardiology Outpatient Clinic of the Faculty of Medical Sciences in Zabrze, Medical University of Silesia in Katowice, Poland. The study was approved by the Bioethics Committee (Medical University of Silesia). The data were collected from October 2016 to September 2023. Patients with SLE were enrolled. An age- and gender-matched control group was studied. Patients with SLE in remission and active disease were included in the study. Patients with other autoimmune connective tissue diseases, CVD, cerebrovascular disease, arterial thrombosis, chronic liver disease, chronic kidney disease or other chronic severe diseases were excluded from the study. Pregnant patients were not enrolled. SLE was diagnosed based on the American College of Rheumatology criteria at the Department of Dermatology in Zabrze, Medical University of Silesia in Katowice, Poland. Disease activity was assessed using the Systemic Lupus Erythematosus Disease Activity Index 2000 (SLEDAI-2K). Scores ≥ 6 indicated active disease.

Body mass index (BMI) was calculated according to the following formula: the ratio of body weight (kg) to the square of height (m^2^). Transthoracic echocardiography (TTE) was performed with a 3.6 MHz probe (GE Healthcare VIVID T9 ultrasound system, USA). Measurements of the left ventricular ejection fraction (LVEF) were performed based on the apical four-chamber and two-chamber views using the modified Simpson method. The results are the mean of at least three LVEF measurements and are expressed as percentages (%). 

CIMT measurements were performed using ultrasound (GE Healthcare VIVID T9) with a 10 MHz linear probe and according to the guidelines of the American Society of Echocardiography (ASECHO). The carotid plaque score (cPS) was calculated by the sum of each segment of the carotid artery with an atherosclerotic plaque (internal carotid artery, external carotid artery and carotid artery bulb). The minimum score was “0” and the maximum score was “6”.

PWV was measured using Complior Analyze (Alam Medical, Saint-Quentin-Fallavier, France). Blood pressure was assessed using the Microlife WatchBP (Microlife AG Swiss Corp., Widnau, Switzerland) according to the manufacturer’s instructions. ABI was measured using Microlife WatchBP Office (Microlife Corporation) [25].

In addition, the distance in the 6 min walk test (m) was assessed. We also evaluated the metabolic equivalent (METs) based on the electrocardiographic exercise test according to the Bruce protocol.

The following tests were performed in the laboratory: lipid profile (total cholesterol [TC], high-density cholesterol [HDL], low-density cholesterol [LDL] and triglycerides [TG]), glucose, C-reactive protein (CRP), creatinine, estimated glomerular filtration rate (eGFR), high-sensitivity cardiac troponin T (hs-cTn), N-terminal prohormone of brain natriuretic peptide (NT pro-BNP), creatine kinase–MB (CK-MB) and D-Dimers. The measurements were performed under the same conditions in the outpatient setting as part of routine patient evaluation at the Cardiology Outpatient Clinic. 

### Statistical Analysis 

Statistical analysis was performed using Statistica version 14.0 (StatSoft Inc., Tulsa, OK, USA). Descriptive data were presented as mean ± standard deviation (SD) or the number and frequency, where appropriate. The type of distribution of selected parameters was analyzed using the Shapiro–Wilk test. The Student’s *t*-test was used for data with normal distribution. The Mann–Whitney U-test was applied for non-parametric data. The unpaired Student’s *t*-test and the analysis of variance (ANOVA) were used to compare the mean values. The independence of categorical variables was assessed using the chi-square test. The correlation coefficient for quantitative variables was assessed using Pearson’s correlation test (for normal distribution) and Spearman’s test (for non-parametric data). A *p*-value < 0.05 was considered statistically significant.

## 3. Results

Ninety-eight patients with SLE were enrolled (87 women, mean age 59 years; 11 men, mean age 60 years). An age- and gender-matched control group consisted of 68 healthy adults, including 60 women (mean age 54 years) and 8 men (mean age 57 years). SLE disease activity was assessed using the SLEDAI-2K index. At enrollment, the patients did not present with heart disease. Anthropometric features showed no significant differences. A larger proportion of patients with SLE were current or former tobacco users. However, no statistical significance was observed. The lipid profile and basic biochemical tests showed no significant differences between the groups. NT proBNP, hs-cTn, CK-MB and D-Dimers were significantly elevated in the SLE group. The parameters were not associated with acute conditions, i.e., acute coronary syndrome, heart failure or thrombosis. The median SLEDAI-2K score was 6 at the time of examination. Higher BMI values in SLE correlated with higher levels of glucose (r = 0.2729, *p* = 0.0489) and LDL (r = 0.3690, *p* = 0.0501). The lipid profile was statistically significant for TG in the study group. There were no significant differences in glucose, CRP or renal parameters. The baseline characteristics of the study group are given in Table 1. 

**Figure 1:**
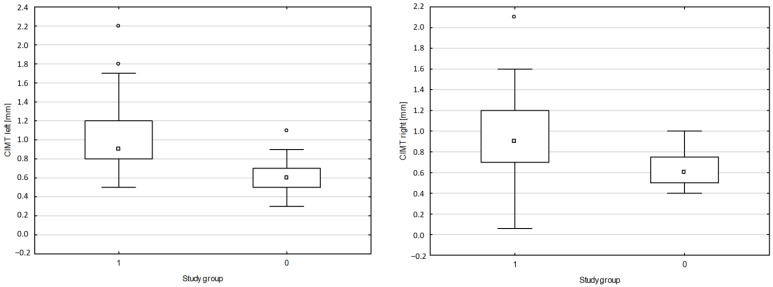
Comparison of CIMT for the right and left carotid arteries in the study group. CIMT: Intima-media complex thickness; □ median; ▭ 25–75%; non-outlier range; ◦ outlier; 1-SLE; 2-Non-SLE. Source: authors’ own research.

**Figure 2:**
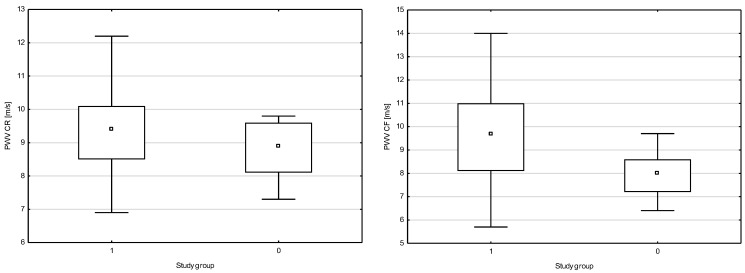
Comparison of PWV CF and PWV CR in the study group. PWV CF: carotid–femoral pulse wave velocity; PWV CR: carotid-radial pulse wave velocity; □ median; ▭ 25–75%; non-outlier range; 1-SLE; 2-Non-SLE. Source: authors’ own research.

**Figure 3:**
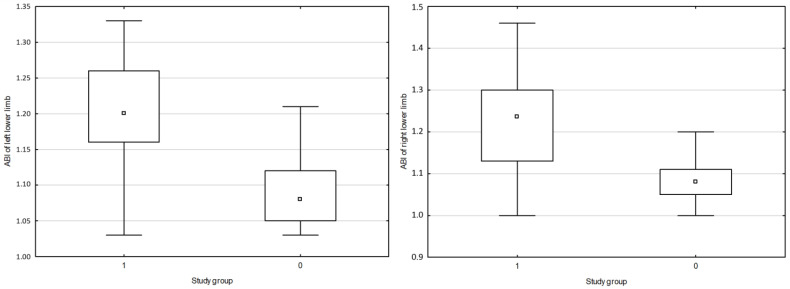
Comparison of ABI for the right and left lower limbs in the study group. ABI: ankle–brachial index; □ median; ▭ 25–75%; non-outlier range; 1-SLE; 2-Non-SLE. Source: authors’ own research.

A positive correlation was found between NT proBNP and PWV CF (r = 0.6880, *p* = 0.0498) and between hs-cTn and PVW CF (r = 0.8862, *p* = 0.0499) in SLE. A positive correlation was observed between PWV CF and systolic blood pressure (BPs) (r = 0.5025, *p* = 0.0487). A negative correlation was reported between NT proBNP and LVEF (r = −0.3826, *p* = 0.0511) in SLE. No difference or correlation was found among the results for PWV CF, CIMT and ABI in the group with active and inactive SLE according to the SLEDAI-2K scale. The data is summarized in Figure 1, Figure 2 and Figure 3.

A positive correlation was found between treatment time and the augmentation index (Alx) (r = 0.5957, *p* = 0.0488). The index correlated negatively with the distance in the 6 min walk test (r = −0.5272, *p* = 0.0498) and METs (r = −0.6479, *p* = 0.0508). A positive correlation was observed between Alx and treatment time in active SLE (r = 0.6833, *p* = 0.0489). A negative correlation was reported between CIMT and ABI (r = −0.6436, *p* = 0.0459) and METs (r = −0.3231, *p* = 0.0512). No significant correlations were found between PWV CR and NT proBNP, hs-cTn, D-dimers and CK-MB. The Factors under study related to the assessment of subclinical atherosclerosis in the study group are given in Table 2.

New-onset CVD was diagnosed—hypercholesterolemia (TC > 190 mg/dL and LDL > 130 mg/dL)—in 26% of patients with SLE and 4% of the controls, hypertension (5% vs. 4%), deterioration of renal function (6% vs. 0%) and deterioration of the left ventricular systolic function (10% vs. 1%). 

## 4. Discussion

The assessment and monitoring of subclinical atherosclerosis is one of the aims of faster CVD diagnosis. In late-stage SLE, a significant percentage of patients die mainly due to CVD. Atherosclerosis is an important line of research in this group of patients. Endothelial dysfunction is a major factor in the early development of atherosclerosis in patients with SLE [5]. According to the European Alliance of Associations for Rheumatology (EULAR), CVD assessment in patients with autoimmune connective tissue disease should be performed at least once every five years and after any major change in anti-inflammatory treatment, and the SCORE result should be multiplied by 1.5. Recommendations include the assessment of subclinical atherosclerosis using arterial Doppler ultrasound (CIMT assessment) [26,27,28]. Of note, CIMT allows the assessment of atherosclerosis only in one narrow vascular segment, while the remaining vascular bed is not assessed. Arterial stiffness results from thickening of the arterial walls and early mechanical changes of the arterial wall. PWV and Alx are non-invasive methods for assessing peripheral and central atherosclerosis, being the gold standard for assessing subclinical atherosclerosis, thus allowing the prediction of future CVD events [29] and assessment of a larger vascular segment. According to the Framingham study, patients with SLE with low CVD risk presented with increased arterial stiffness [8].

The results of this study provide evidence that the presence of subclinical atherosclerosis in SLE does not depend only on the occurrence of severe organ complications. Our finding confirms that arterial abnormalities are already present in asymptomatic patients. Arterial abnormalities were observed based on increased CIMT, the presence of atherosclerotic plaque and increased arterial stiffness. Our study showed significant correlations between biochemical parameters and arterial stiffness, which was common to CIMT and ABI as established diagnostic tests.

Systemic lupus erythematosus is a disease that can occur at an early age, thus determining the patient’s future and increasing the risk of CVD. Studies confirmed that subclinical atherosclerosis can occur as early as adolescence [9,10,11]. Barsalou et al. studied adolescent patients with SLE (*n* = 149) compared to healthy controls. They showed that CIMT, flow-mediated dilation (FMD) and PWV scores did not differ from healthy controls. However, FMD worsened with disease duration, which may indicate progressive endothelial dysfunction already at a young age [30], which is in line with our findings. In our study, significantly worse PWV, CIMT or ABI were found in adult patients with SLE compared to healthy controls. It seems that arterial stiffness is age-related, and significantly abnormal results are found in the elderly [11]. Su-angka et al. assessed 102 patients with SLE and 103 healthy controls. Median CIMT was not significantly different in all groups. Higher carotid arterial stiffness index (CASI) in active and inactive SLE in children, suggesting functional changes in the carotid arteries, may be early evidence of increased atherosclerosis in pediatric patients with SLE. This functional dysfunction was found active and in-remission SLE [8]. Atkinson et al. found that PWV was elevated in a group of children with SLE with renal involvement [31], which may be associated with significant future exposure to CVD in these patients. Impaired renal function is associated with vascular calcification, even in the early stages of kidney disease, and may be associated with increased arterial stiffness [32]. In the study group, no correlation was found between renal function and arterial stiffness. However, the values were within normal limits, or mild renal impairment was found (eGFR 46–59 mL/min/1.73 m^2^). Of note, PWV is independently associated with age, MAP and eGFR in patients with SLE [33]. Morreale et al. showed similar results to those obtained in our study for the control and study groups (PWV 7.1 vs. 9.1; CIMT 0.56 vs. 0.81) [34]. Similar results were also obtained in other studies [4,25,33,35,36,37,38,39]. Sacre et al. observed an increase in arterial stiffness in patients with SLE, which corresponded to subclinical atherosclerosis as assessed by CIMT, and PWV CF correlated with CIMT [8], which was also confirmed in our study. However, we extended the correlation by biochemical parameters. Roldan et al. and Parra et al. showed similar results to ours—CIMT, atherosclerosis and abnormal arterial stiffness were more prevalent in patients with SLE than in controls [40,41]. However, in a study by Tziomalos et al., PWV (6.3 vs. 7.3 m/s) and ABI (1.04 vs. 1.10) were lower in patients with SLE [42]. Of note, the study group was twice as small as ours. Unfortunately, the small study group is a major limitation of most studies. It is possible that in the control group, more smokers influenced the results, and CVD was not excluded and the ABI test was not performed separately for each lower limb but only for one limb. Also, Stortz et al. obtained non-statistically significant results for PWV (6.71 vs. 6.40 m/s). The SLE group included 125 patients in remission (mean age 46 years). Patients being treated for hypertension were included in the study. Blood pressure in the SLE group was significantly higher than in the control group (19.2% vs. 36%). A similar situation was observed in the case of type 2 diabetes mellitus (0% vs. 9%), which might have significantly influenced the results [43]. Of note, a Vicorder with two cuffs around the neck and thigh was used. In our study, the Complior system was applied. It includes two piezoelectric sensors recommended by the European Society of Hypertension [44]. Abnormal blood pressure and diabetes can cause an increase in PWV. In our study, the study group was older. We did not include patients with previously diagnosed CVD and diabetes. However, CVD was diagnosed during the study. Age, hypertension and impaired renal function influence increased arterial stiffness in SLE [8,32,42,43,45,46,47]. Analysis of PWV, regardless of the artery, showed a significantly higher mean (1.12 m/s) in 1234 patients with SLE compared to 678 controls [4]. Compared to controls, a significant increase in central PWV was found in 777 patients with SLE [33,36,37,38,39,48,49], which was also confirmed in our study. The data collected from 78 patients with SLE and 188 healthy controls showed that peripheral PWV was higher in patients with SLE [50,51]. Of note, their study group was smaller compared to ours. We did not find any significant differences in PWV CR, which may be related to a different vascular structure than in the case of the central vascular bed. 

Patients with SLE who achieved remission or presented with a lower SLEDAI score showed improvement in arterial stiffness [52]. It was also confirmed that higher CIMT correlated with (very) high levels of disease activity [53]. In our study, no statistically significant difference was found in outcomes between active disease and the remission of SLE. However, no prospective studies were performed. Therefore, the early prediction of CVD is very important for adequate treatment and long-term follow-up of patients with SLE. Several studies evaluating the augmentation index showed significantly higher values in patients with SLE compared to controls and significant heterogeneity between studies. Increased arterial stiffness and endothelial dysfunction were observed in patients with SLE without CVD and significant atherosclerotic lesions [36,37,41,50,51,54,55,56]. The results of these studies support our findings. We showed that Alx significantly correlated with disease duration.

A correlation was found between PWV measurements and biochemical biomarkers, such as the B-type natriuretic peptide [57], which was also confirmed in our study with a cutoff point for the diagnosis of heart failure. Sabio et al. evaluated the correlations between high-sensitivity cardiac troponin I (hs-cTnI) and PWV CF. Their study found that the SLE group had higher levels of hs-cTnI compared to the control group, while PWV showed no significant differences between the groups [58]. Similar results were obtained in two studies where PWV values correlated with higher hs-cTnI [19,58]. However, PWV was higher in the SLE group. Of note, their study group was much younger than ours. Elevated hs-cTn below the cutoff point for the acute coronary event correlated with the presence of subclinical atherosclerosis and atherosclerotic plaques in other studies [21,59,60]. In our study, we evaluated hs-cTn, which was significantly higher in the SLE group, but it did not meet the criteria for the diagnosis of acute coronary syndrome and correlated with higher PWV, which may indirectly indicate ongoing atherogenesis. Other studies assessed troponin I, while we used troponin T, which was available in our laboratory. Of note, there have been few studies on the relationship between hs-cTn and PWV and the cutoff point that can be applied in clinical practice. Therefore, this aspect requires further research.

Studies demonstrated that arterial stiffness is also associated with increased concentric left ventricular remodeling [61]. In our study, we assessed the left ventricular ejection fraction, which showed no significant correlation with PWV. Further studies on this issue are warranted with more echocardiographic parameters. Montalbán-Méndez et al. assessed the 6 min walk test, which negatively correlated with PWV in a cohort of 49 patients with SLE [62]. In the study group, we did not show such a correlation. However, Alx correlated negatively with the distance in the 6 min walk test and METs in the exercise test. We also found a significant difference in distance compared to the controls. 

Our study is also clinically relevant. During routine assessment, potentially healthy individuals were diagnosed with new-onset CVD (hypercholesterolemia, hypertension, deterioration of renal function and deterioration of the left ventricular systolic function). After excluding new-onset CVD, the results did not change significantly. However, the above conditions were diagnosed at an early stage.

### Study Limitations 

Our study has some limitations. The limited sample size was considered an important factor. Although SLE is a rare disease in Poland, a group of about 100 patients is still a small sample compared to multicenter studies. Patients with previously diagnosed CVD (i.e., hypertension, ischemic heart disease or heart failure) were not included in the study. The enrollment of such patients would have made predictions regarding atherosclerosis difficult in some groups of patients. The baseline patient characteristics may explain the results of our study, such as newly diagnosed CVD. Our study did not consider SLE treatment. Studies on this issue were published in other papers we referred to so as not to duplicate the data [25,63,64]. There is a need to develop an abbreviated protocol for implementation in daily practice.

## 5. Conclusions

Further studies are warranted to establish the prognostic value of PWV and biochemical markers in patients with SLE for early diagnosis of subclinical atherosclerosis. PWV, which is used to assess a larger vascular segment, is significantly superior to the assessment with CIMT alone. However, both examinations provide a reproducible, non-invasive assessment that is desirable in daily practice, and they may be complementary. Our study provides a basis for further research related to endothelial dysfunction and subclinical atherosclerosis in SLE and assessment of the predictive value of PWV in CVD risk.

## Figures and Tables

**Table 1:** Demographic, clinical and serological characteristics of the study group.

Patient Characteristics	Study Group Mean ± SD	Control Group Mean ± SD	*p* Value
Age (years)	59.89 ± 11.47	54.86 ± 10.87	0.8116
Female (%) (*n*)	89 (87)	88 (60)	0.9448
BMI	26.27 ± 4.77	27.16 ± 4.99	0.3992
Smokers			
Current (%) (*n*)	14 (14)	5 (7)	0.5119
Former (%) (*n*)	20 (20)	5 (7)	0.0997
Disease duration (years)	10.55 ± 7.93		
Lipid profile			
TC (115.00–190.00 mg/dL)	197.08 ± 47.15	199.78 ± 39.69	0.7920
HDL (>40 mg/dL)	61.85 ± 21.03	67.56 ± 15.94	0.2306
LDL (<115 mg/dL)	120.49 ± 42.11	101.97 ± 32.95	0.0663
TG (<150 mg/dL)	120.33 ± 48.69	93.29 ± 26.91	**0.0092**
Glucose (70–99 mg/dL)	92.50 ± 13.17	92.07 ± 13.69	0.8813
Creatine (62–106 μ ol/L)	68.10 ± 29.31	64.77 ± 11.76	0.5678
eGFR (>90 mL/min/1.73 m^2^)	75.78 ± 21.42	83.06 ± 7.94	0.2514
CRP (<5 mg/L)	9.74 ± 40.48	2.44 ± 3.05	0.4749
NT proBNP (0.00–125.00 pg/mL)	144.87 ± 183.26	36.41 ± 14.58	**0.0018**
hs-cTn (<14 ng/L)	25.43 ± 27.13	6.38 ± 2.93	**0.0303**
CK-MB (<12 j.m/L)	28.89 ± 8.00	16.01 ± 3.15	**0.0002**
D-Dimer (<500 µg/mL)	0.73 ± 0.28	0.36 ± 0.18	**0.0088**
Serological % (*n*)			
ANAs	90 (88)		
dsDNA	53 (52)		
RNP	7 (7)		
SSA/Ro	40 (39)		
SSB/LA	8 (8)		
Sm	21 (21)		
SLEDAI-2K (point)	8.18 ± 7.78		

BMI: body mass index; TC: total cholesterol; HDL: high-density cholesterol; LDL: low-density cholesterol; TG: triglycerides; eGFR: estimated glomerular filtration rate; CRP: C-reactive protein; NT proBNP: N-terminal prohormone of brain natriuretic peptide; hs-cTn: high-sensitivity cardiac troponin T; CK-MB: creatine kinase–MB; ANAs: antinuclear antibodies; dsDNA: anti-double-stranded DNA antibodies; RNP: anti-ribonucleoprotein antibodies; SSA/Ro: anti-Ro antibodies; SSB/LA: anti-La antibodies; Sm: anti-Smith antibodies; SLEDAI-2K: Systemic Lupus Erythematosus Disease Activity Index. Bold is statistics important. Source: authors’ own research.

**Table 2:** Factors under study related to the assessment of subclinical atherosclerosis in the study group.

Parameters	Study Group (SLE) Mean ± SD	Control Group Mean ± SD (under Control Group)	*p* Value
CIMT left [mm]	1.03 ± 0.43	0.62 ± 0.20	**<0.0001**
CIMT right [mm]	0.93 ± 0.34	0.63 ± 0.16	**<0.0001**
cPS % (*n*)	19 (19)	5 (7)	**<0.0001**
ABI right ankle	1.22 ± 0.12	1.09 ± 0.07	**0.0107**
ABI left ankle	1.21 ± 0.07	1.10 ± 0.07	**0.0021**
PWV CF [m/s]	9.74 ± 2.22	7.98 ± 1.14	**0.0294**
PWV CR [m/s]	9.34 ± 1.42	8.78 ± 0.97	0.2995
Peripheral BP systolic [mmHg]	128.67 ± 13.23	125.33 ± 11.61	0.5274
Peripheral BP diastolic [mmHg]	78.72 ± 7.28	80.22 ± 4.89	0.5834
HR [bpm]	66.72 ± 11.32	71.00 ± 5.31	0.2956
Central BP systolic [mmHg]	118.94 ± 14.53	117.33 ± 9.55	0.7666
Central BP diastolic [mmHg]	78.72 ± 7.28	80.22 ± 6.03	0.5995
Alx [%]	23.30 ± 12.12	9.80 ± 14.72	**0.0193**
LVEF [%]	54.21 ± 6.41	57.33 ± 5.24	**0.0442**
6MTW [m]	514.26 ± 84.80	614.03 ± 88.01	**<0.0001**
METs	8.84 ± 2.29	11.30 ± 1.63	**<0.0001**

SD: standard deviation; CIMT: intima-media complex thickness; cPS: carotid plaque score; ABI: ankle–brachial index; PWV CF: carotid–femoral pulse wave velocity; PWV CR: carotid–radial pulse wave velocity; BP: blood pressure; HR: heart rate; Alx: augmentation index; LVEF: left ventricular ejection fraction; 6MTW: six-minute walk test; METs: metabolic equivalent. Bold is statistics important. Source: authors’ own research.

## Data Availability

Data are included in the paper. Some data were reported in an abstract for the ESC Preventive Cardiology Congress, Athens, Greece (25–27 April 2024). While submitting the paper for publication, we were waiting for the acceptance of the abstract to present our research.
